# Species diversity analysis of commercial Mantidis Ootheca samples contaminated by store pests based on DNA metabarcoding

**DOI:** 10.1186/s12864-022-08955-1

**Published:** 2022-10-21

**Authors:** Liuwei Xu, Xiaoying Zhang, Hua Guo, Xia Yang, Zhimei Xing, Wenzhi Yang, Jian Zhang, Xiaoxuan Tian

**Affiliations:** 1 State Key Laboratory of Component-based Chinese Medicine, Tianjin University of Traditional Chinese Medicine, Tianjin, 301617 China; 2 Haihe Laboratory of Modern Chinese Medicine, Tianjin, 301617 China; 3 School of Chinese Materia, Tianjin Universtity of Traditional Chinese Medicine, Tianjin, 301617 China

**Keywords:** Mantidis Ootheca, Sangpiaoxiao, DNA metabarcoding, mini-barcode

## Abstract

Mantidis Ootheca (Sangpiaoxiao, mantis egg case) is a typical multi-origin Chinese medicinal material. The Chinese Pharmacopoeia stipulates that the Mantidis Ootheca originates from three species of Mantis: *Tenodera sinensis*, *Statilia maculate*, and *Hierodula patellifera*. However, Mantidis Ootheca mainly relies on field collection, which leads to confusion of its actual origin in the market. As the clinical use of Mantidis Ootheca with unknown original mantis species will pose potential risks to drug safety, it is necessary to survey the commercially available Mantidis Ootheca origin species. However, as the egg case of Mantis, the morphological characters of Mantidis Ootheca are limited and usually cannot serve as accurate identification tool. DNA barcoding, which is widely used in taxonomic studies of animals, is severely affected by the impact of storage pests and DNA degradation. Thus, this study collected a total of 4580 Mantidis Ootheca and pooled separately Mantidis Ootheca samples according to 18 different sources as DNA samples to analyze the origin diversity of Mantidis Ootheca individuals contaminated by common store pests collected in in the market using DNA metabarcoding, and to provide a basis for quality control of Mantidis Ootheca. 37 Mantis ASVs and 9 Mantis MOTUs were identified through species delimitation, and the high-level intraspecific diversity was depicted as haplotype network plot. Besides *Tenodera sinensis* and *Hierodula patellifera* as genuine original mantis species defined in the Chinese Pharmacopoeia, *Tenodera angustipennis* was also the origin species of these Mantidis Ootheca samples.

**Supplementary Information:**

The online version contains supplementary material available at 10.1186/s12864-022-08955-1.

## Introduction

Mantidis Ootheca is the egg case of Mantis, which is one of the traditional Chinese medicine (TCM) originally recorded in “Shen Nong Ben Cao Jing” [1]. Modern research has shown that Mantidis Ootheca includes N-acetyldopamine derivatives, which has significant antioxidant activity [2]. The Chinese Pharmacopoeia classifies Mantidis Ootheca into three authentic varieties: Tuanpiaoxiao, Changpiaoxiao, and Heipiaoxiao, with the origin mantis species corresponding to *Tenodera sinensis*, *Statilia maculate*, and *Hierodula patellifera*, respectively [3]. However, Mantidis Ootheca relies mainly on field collection. As there are at least 112 species (including subspecies) of Mantis in China [4], the Mantis egg cases which were not stipulated in the Chinese Pharmacopoeia may be involved as Mantidis Ootheca. For instance, Wen et al. [5] and Wang et al. [6] found that *Statilia nemoralis* and *Mantis religiosa* were also the origin species of Mantidis Ootheca. Moreover, *Titanodula menglaensis sp* as a newly described species in the Mantis subfamily Hierodulinae [7], indicated the existence of cryptic species in Mantis. Since Mantidis Ootheca of different origin species may have different pharmacological effects [8], accurate identification of Mantidis Ootheca is particularly important to ensure clinical safety.

Macroscopical identification has been widely used for TCM authentication. However, as the morphological features of oothecae have not yet been thoroughly studied in Mantis, origin species identification of Mantidis Ootheca is often difficult because of insufficient classification background and ambiguous morphological characteristics. Recently, DNA barcoding has been generally accepted as an effective tool for rapid, accurate species-level identifications and for the discovery of cryptic species [9, 10]. Nevertheless, due to the technique basis of “one-by-one DNA barcoding” method using Sanger sequencing of a PCR amplicon from an individual organism, it is usually difficult to identify animal samples with disturbance of storage pests, without laborious steps of cloning of PCR products. Moreover, with regard to samples in bulk like commercial herbal materials, traditional DNA barcoding can be prohibitively expensive and laborious, and often susceptible to DNA degradation during storage.

Nowadays, DNA metabarcoding, the coupling of DNA barcoding with high-throughput sequencing, enables the analysis of a large number of samples simultaneously. On the other hand, DNA mini-barcodes, short DNA sequences of 100–250 bp, with sufficient variable sites could be a solution to overcome the difficulties of DNA degradation. In this study, DNA metabarcoding combined with DNA mini-barcode was used to distinguish 4580 Mantidis Ootheca individuals partially disrupted by storage pests, haplotype information at intraspecies level was depicted.

## Material and methods

### Sample collection

A total of 4580 Mantidis Ootheca individuals from 18 different source regions were purchased online and from Anguo Market for Chinese Herbal Medicine, a local market in Hebei (Table 1).

**Table 1:** Sample information

Sample ID	Sampling place/source	Purchase channel	Number of Mantidis Ootheca	Origin species of Mantidis Ootheca in each sample	Storage pests in each sample
spx1	JILIN	ONLINE	160	*Hierodula patellifera*, *Hierodula* sp.*, Tenodera sinensis*, *Tenodera angustipennis*	*Blattisocius tarsalis*, *Stegobium* sp., *Trogoderma variabile*, Dermestidae sp1*.*, Carabidae sp., Chrysomelidae sp1*,* Chrysomelidae sp2, *Lasioderma serricorne*, Torymidae sp.,
spx2	JILIN	ONLINE	240	*Hierodula patellifera*, *Hierodula* sp. *Tenodera sinensis*	*Blattisocius tarsalis*, *Stegobium* sp., *Tribolium castaneum*, *Trogoderma variabile*, Carabidae sp., Chrysomelidae sp1, Chrysomelidae sp2, *Lasioderma serricorne*, Torymidae sp., Tydeidae sp.
spx3	SHANDONG	ONLINE	401	*Hierodula patellifera*, *Hierodula* sp. *Tenodera sinensis*	*Blattisocius tarsalis*, *Stegobium* sp., *Tribolium castaneum*, *Trogoderma variabile*, Dermestidae sp1*.*, Carabidae sp., Chrysomelidae sp1, Chrysomelidae sp2, *Lasioderma serricorne*, Torymidae sp., Tydeidae sp.
spx4	SHANDONG	ONLINE	250	*Hierodula patellifera*, *Hierodula* sp. *Tenodera sinensis*	*Blattisocius tarsalis*, *Stegobium* sp., *Tribolium castaneum*, *Trogoderma variabile*, Dermestidae sp1*.*, Carabidae sp., Chrysomelidae sp1, Chrysomelidae sp2, *Lasioderma serricorne*, Torymidae sp., Tydeidae sp.
spx5	GUANGXI	ONLINE	423	*Hierodula patellifera*, *Hierodula* sp. *Tenodera sinensis*	*Blattisocius tarsalis*, *Stegobium* sp., *Tribolium castaneum*, *Trogoderma variabile*, Dermestidae sp1*.*, Carabidae sp., Chrysomelidae sp1, Chrysomelidae sp2, *Lasioderma serricorne*, Torymidae sp., Tydeidae sp.
spx6	GUANGXI	ONLINE	348	*Hierodula patellifera*, *Hierodula* sp. *Tenodera sinensis*	*Blattisocius tarsalis*, *Stegobium* sp., *Tribolium castaneum*, *Trogoderma variabile*, Carabidae sp., Chrysomelidae sp1, Chrysomelidae sp2, *Lasioderma serricorne*, Torymidae sp., *Acarus farris*
spx7	HEBEI	ONLINE	258	*Hierodula patellifera*, *Hierodula* sp. *Tenodera sinensis*	*Blattisocius tarsalis*, *Stegobium* sp., *Tribolium castaneum*, *Trogoderma variabile*, Dermestidae sp1*.*, Carabidae sp., Chrysomelidae sp1, Chrysomelidae sp2, *Lasioderma serricorne*, Torymidae sp., Tydeidae sp.
spx8	SICHUAN	ONLINE	346	*Hierodula patellifera*, *Hierodula* sp. *Tenodera sinensis*	*Blattisocius tarsalis*, *Stegobium* sp., *Tribolium castaneum*, *Trogoderma variabile*, *Trogoderma* sp.*,* Dermestidae sp1*.*, Carabidae sp., Chrysomelidae sp1, Chrysomelidae sp2, *Lasioderma serricorne*, Torymidae sp., Tydeidae sp.
spx9	ANHUI	ONLINE	298	*Hierodula patellifera*, *Hierodula* sp. *Tenodera sinensis*	*Blattisocius tarsalis*, *Stegobium* sp., *Tribolium castaneum*, *Trogoderma variabile*, *Trogoderma* sp.*,* Dermestidae sp1*.*, Carabidae sp., Chrysomelidae sp1, Chrysomelidae sp2, *Lasioderma serricorne*, Torymidae sp., Tydeidae sp.
spx10	HUNAN	ONLINE	235	*Hierodula patellifera*, *Hierodula* sp. *Tenodera sinensis*	*Blattisocius tarsalis*, *Stegobium* sp., *Tribolium castaneum*, *Trogoderma variabile*, *Trogoderma* sp.*,* Dermestidae sp1*.*, Carabidae sp., Chrysomelidae sp1, Chrysomelidae sp2, *Lasioderma serricorne*,
spx11	HENAN	ONLINE	346	*Hierodula patellifera*, *Hierodula* sp. *Tenodera sinensis*	*Blattisocius tarsalis*, *Stegobium* sp., *Tribolium castaneum*, *Trogoderma variabile*, *Trogoderma* sp.*,* Carabidae sp., Chrysomelidae sp1, Chrysomelidae sp2, *Lasioderma serricorne*,
spx12	HENAN	ONLINE	262	*Hierodula patellifera*, *Hierodula* sp. *Tenodera sinensis*	*Blattisocius tarsalis*, *Stegobium* sp., *Tribolium castaneum*, *Trogoderma variabile*, *Trogoderma* sp.*,* Dermestidae sp1., Carabidae sp., Chrysomelidae sp1, Chrysomelidae sp2, *Lasioderma serricorne*, Torymidae sp., *Tyrophagus putrescentiae, Tyrophagus* sp.
spx13	HENAN	ONLINE	365	*Hierodula patellifera*, *Hierodula* sp. *Tenodera sinensis*, *Tenodera angustipennis*	*Blattisocius tarsalis*, *Stegobium* sp., *Tribolium castaneum*, *Trogoderma variabile*, *Trogoderma* sp.*,* Dermestidae sp1., Carabidae sp., Chrysomelidae sp1, Chrysomelidae sp2, *Lasioderma serricorne*, Torymidae sp.,
spx14	ANGUO	Medicinal market	150	*Hierodula patellifera*, *Hierodula* sp. *Tenodera sinensis*, *Tenodera angustipennis*	*Blattisocius tarsalis*, *Stegobium* sp., *Tribolium castaneum*, *Trogoderma variabile*, Dermestidae sp1., Carabidae sp., Chrysomelidae sp1, Chrysomelidae sp2, *Lasioderma serricorne*, Tydeidae sp.
spx15	ANGUO	Medicinal market	150	*Hierodula patellifera*, *Hierodula* sp. *Tenodera sinensis*	*Blattisocius tarsalis*, *Stegobium* sp., *Tribolium castaneum*, *Trogoderma variabile*, *Trogoderma* sp.*,* Dermestidae sp1., Carabidae sp., Chrysomelidae sp1, Chrysomelidae sp2, *Lasioderma serricorne*, Torymidae sp., Dermestidae sp2. *Tetraneura* sp.
spx16	ANGUO	Medicinal market	153	*Hierodula patellifera*, *Hierodula* sp. *Tenodera sinensis*	*Blattisocius tarsalis*, *Stegobium* sp., *Tribolium castaneum*, *Trogoderma variabile*, *Trogoderma* sp.*,* Dermestidae sp1., Carabidae sp., Chrysomelidae sp1, Chrysomelidae sp2, *Lasioderma serricorne*, Torymidae sp., Tydeidae sp.
spx17	ANGUO	Medicinal market	117	*Hierodula patellifera*, *Hierodula* sp. *Tenodera sinensis*	*Blattisocius tarsalis*, *Stegobium* sp., *Trogoderma variabile*, Carabidae sp., Chrysomelidae sp1, Chrysomelidae sp2, *Lasioderma serricorne*, Torymidae sp., Tydeidae sp.
spx18	ANGUO	Medicinal market	78	*Hierodula patellifera*, *Hierodula* sp. *Tenodera sinensis*	*Stegobium* sp., *Tribolium castaneum*, *Trogoderma variabile*, Carabidae sp., Chrysomelidae sp1, Chrysomelidae sp2, *Lasioderma serricorne*, Tydeidae sp.

### Macroscopical identification and DNA barcoding of ten Mantidis Ootheca representative samples

As a pilot investigation, ten Mantidis Ootheca with typical different shapes and colors were selected from all individuals (Supplementary Table S1).

According to the protocol of DNA barcoding, approximately 0.01 g of internal tissue was cut from each Mantidis Ootheca sample and lysed with 1.5% sodium dodecyl sulfate [11]. The mixture was incubated at 65 °C for 20 minutes in a water bath and centrifuged at 3000 rpm (revolutions per minute) for 5 minutes. Genomic DNA was extracted from 200 μl of supernatant with the TIANamp Genomic DNA Kit (Tiangen Biotech Co., Ltd., Beijing, China) according to the manufacturer’s instructions. DNA extractions were carried out in a dedicated pre-PCR laboratory. The equipment and workstation wiped with 75% ethanol and then sterilized by UV lamps [12]. Forward primer LCO1490 (5′-GGTCAACAAATCATAAAGATATTGG-3′), and the reverse primer HCO2198 (5′-TAAACTTCAGGGTGACCAAAAAATCA-3′) were used to amplify the region of cytochrome c oxidase subunit I (COI) [13]. PCR reactions were carried out in a volume of 50 μl containing 5 μl 10 × Fast Buffer I (Takara Dalian, China), 4 μl dNTP, 0.25 μl SpeedSTAR™ HS DNA Polymerase (Takara Dalian, China), 2 μl DNA template, 1 μl for each primer (final concentration 0.2 μM), and 36.75 μl ddH_2_O. The following thermal cycling conditions were applied: initial denaturing at 98 °C for 1 minute, followed by 30 cycles of 98 °C for 5 seconds, 48 °C for 15 seconds, 72 °C for 8 seconds with a final extension at 72 °C for 5 minutes. The negative PCR controls were analyzed in parallel to the samples to monitor possible contaminations during the PCR step. PCR products (including negative controls) were separated on 2% agarose gels at 110 V for 30 minutes and stained by ethidium bromide to determine the length of the amplified product fragments. The PCR product was sequenced with an ABI Prism 3730 sequencer (Applied Biosystems). The COI sequences were edited using Geneious version 8.0.4. The resulting sequences were blasted and evaluated on coverage, E-value and % match against the NCBI GenBank database.

### DNA metabarcoding analysis of all Mantidis Ootheca samples

#### Mini-barcode performance in silico

Metabarcoding studies on bulk collections of animals usually target a subset of the 658 bp COI “Folmer” region [13–15]. Therefore, we used the forward primer LCO1490, and the reverse primer HCO1777 (5′-ACTTATATTGTTTATACGAGGGAA-3′) to amplify a 232 bp fragment on the COI gene [16]. To evaluate the discriminatory ability of the primers on Mantis, the related COI sequences from NCBI were downloaded, and the 658 bp and 232 bp sequence matrix were used to construct phylogenetic tree based on neighbor-joining (NJ) method in MEGA V.11.0.1, respectively.

#### DNA extraction and PCR amplification

All individuals according to 18 different sources were pooled separately as DNA samples for DNA extraction and PCR amplification. Approximately 0.01 g of internal tissue was cut from each individual Mantidis Ootheca and collected into the corresponding 18 test tubes according to the place of origin. Then 1.5% sodium dodecyl sulfate was added at a ratio of 1:8 for lysis. The next DNA extraction and PCR amplification operations were the same as above with the TIANamp Genomic DNA Kit (Tiangen Biotech Co., Ltd., Beijing, China) according to the manufacturer’s instructions. To distinguish multiple samples simultaneously after sequencing, both LCO1490/ HCO1777 primers were tagged with unique 8 bp tags at the 5′ end (Supplementary Table S2). Subsequently, PCR products were mixed in equimolar amounts in a dedicated no-DNA laboratory to minimize the risk of contamination. The sequencing library was generated using a NEBNext® Ultra™ DNA Library Prep Kit for Illumina (NEB, USA) following the manufacturer’s recommendations. Sequencing was performed on the Illumina NovaSeq platform NovaSeq 6000, and 2 × 250 bp paired-end reads were generated. Sequencing run volume of 2.5 G of data, and returned 5 million sequences.

#### Sequence analysis

The raw reads were first cleaned by removing adapter sequences, trimming low-quality ends, and filtering reads with low quality (Phred quality < 20) using Trimmomatic [17]. Sequencing reads were demultiplexed using fastq-multx and assigned to each sample according to the unique tags [18]. Primer and tag sequences were trimmed using bbduk from BBMap tools [19]. The parameters were set as: k = 15, mink = 2, ktrim = l, minlength = 180, maxlength = 240. Overlapping paired-end reads were merged using fastq-join and were processed with QIIME V.1.9 [20]. A quality check of Q > 30 was performed on the merged fastq data. We then dereplicated reads using the USEARCH [21, 22] fastx_uniques algorithm, with the parameter minuniquesize 2. We applied the USEARCH UNOISE3 algorithm to detect and remove chimeras with the default parameters, substitutions due to incorrect base calls and gaps due to omitted or spurious base calls. The 232 bp amplicon sequences were retained using akutils-v1.2. USEARCH was used to cluster amplicon sequencing variants (ASVs) at a 100% similarity threshold. ASVs with a relative abundance of less than 0.01% of total reads were removed using QIIME. Representative nucleotide sequences from ASVs were imported into Geneious Prime 2020.2. These sequences were aligned using MAFFT v7.017. The genetic code was Invertebrates Mitochondrial and chose the appropriate frame for translation. These sequences were translated into amino acid sequences and any sequences that contained stop codons were removed, and then, the ASV table and representative sequences were regenerated. BLASTN [23] was used to compare the ASV representative sequences against the NCBI GenBank database, and the output was imported into MEGAN version 6.10.8 [24]. MEGAN parameters were set as: minimum score = 50, maximum expected = 0.01, top percent = 10, minimum support percent = 0.01, minimum support = 1 and weighted LCA algorithm. Species-level taxonomy was assigned when the identity values between the query and reference sequences were above 98% [25]. The minimum identity to query would be set as 92% to obtain taxonomic information at a higher level for queries which could not be identified as exact species. The read counts and Mantis read coverage (Mantis reads/number of Individual) for each sample were recorded.

#### Species-delimitation and haplotype-network analysis

For ASVs without species level information, Automatic Barcode Gap Discovery method (ABGD) and Bayesian implementation of the Poisson tree processes model (bPTP) were used for species delimitation. ABGD was conducted on the webserver (https://bioinfo.mnhn.fr/abi/public/abgd/abgdweb.html) using Kimura (K80) TS/TV model to calculate the genetic distances. The Bayesian tree was built under the GTR + F + I model (obtained by ModelFinder) in PhyloSuite v1.2.2. The Bayesian tree was uploaded to the bPTP web server (https://species.h-its.org) for estimating species formation and branching events, with 500,000 MCMC generations, 100 thinnings, and burn-in of 0.1.

For further genetic relationship analysis of Mantis ASVs, COI sequences of four genuine Mantidis Ootheca origin mantis species as *Tenodera sinensis*, *Statilia maculate*, *Hierodula patellifera*, and *Tenodera angustipennis* were obtained from GenBank, and then aligned with our Mantis ASVs to construct a data matrix. The details of these sequences’ information were shown in Supplementary Table S3. Haplotype data was generated using DnaSP V.6.12.03, and then the TCS haplotype network was generated using PopART v 1.7.

## Results

### Sample collection

The information of 4580 Mantidis Ootheca individuals from the 18 samples were in Table 1. Origin species of Mantidis Ootheca and storage pests in each sample were determined by DNA metabarcoding in this study. The 18 samples were sourced from eight provinces (JILIN, SHANDONG, GUANGXI, HEBEI, SICHUAN, ANHUI, HUNAN and HENAN) and one Chinese herbal market (ANGUO) in China.

### Identification of ten Mantidis Ootheca samples with representative morphological characters

Morphologically, the oothecas of *Hierodula patellifera* (Heipiaoxiao) are ellipsoid in shape and black in colour; those of *Tenodera sinensis* (Tuanpiaoxiao) are barrel-like in shape and yellow-brown in colour; and those of *Tenodera angustipennis* are fusiform in shape and brown in colour.

**Fig. 1:**
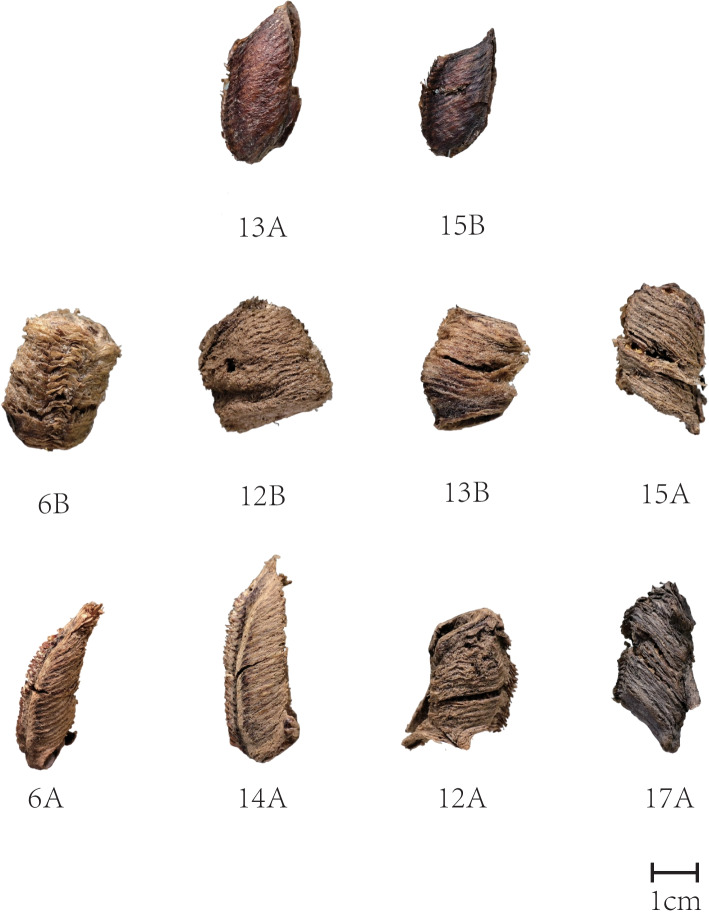
The morphology of Ten Mantidis Ootheca representative samples. 13A and 15B were identified as *Hierodula patellifera* (Heipiaoxiao). 6B, 12B, 13B, and 15A were regarded as *Tenodera sinensis* (Tuanpiaoxiao). 6A, 12A, 14A, and 17A were determined as *Tenodera angustipennis*

According to the macroscopical characteristics of 10 Mantidis Ootheca representative samples, 13A and 15B were identified as from *Hierodula patellifera* (Heipiaoxiao), while 6B, 12B, 13B, and 15A were regarded as ootheca of *Tenodera sinensis* (Tuanpiaoxiao), and the morphology of sample 6A, 12A, 14A, and 17A were consistent with that of *Tenodera angustipennis*, as a common adulterant of Mantidis Ootheca. However, as illustrated in Fig. 1, there were still morphological variances within each designated species, and further DNA barcoding was applied to verify our species identification and uncover intraspecific biodiversity.

### DNA barcoding identification of ten Mantidis Ootheca representative samples

Surprisingly, although COI region data from all of ten individuals were successfully obtained (Table 2), only four of them were distinguished as Mantis species, including 6B and 13B as *Tenodera sinensis* (Tuanpiaoxiao), 13A as *Hierodula patellifera* (Heipiaoxiao), and 14A as *Tenodera angustipennis*, which was in agreement with the results of morphological identification. For the other samples, five amplicon sequences were identified as common stored product pests, as *Acarus farris* (Acaridae), *Tyrophagus putrescentiae* (Acaridae, cereal mite), *Dermestes coarctatus* (Dermestidae) and *Tetraneura nigriabdominalis* (Eriodomatidae, rice root aphid), and the rest one was identified as from human being. This result could arise for either of two reasons: the contamination from workers and storage pests and the degradation of Mantidis Ootheca DNA.

**Table 2:** DNA barcoding results of ten Mantidis Ootheca representative samples

Sample ID	GenBank Blast Results	Percent Identity (%)	Query coverage (%)	E-value
6A	*Acarus farris*^a^	98.05	80	0.0
6B	*Tenodera sinensis*	99.85	97	0.0
12A	*Tyrophagus putrescentiae*^a^	98.48	99	0.0
12B	*Tyrophagus putrescentiae*^a^	96.52	98	0.0
13A	*Hierodula patellifera*	100	97	0.0
13B	*Tenodera sinensis*	99.24	97	0.0
14A	*Tenodera angustipennis*	99.39	97	0.0
15A	*Dermestes coarctatus*^a^	83.31	99	0.0
15B	*Tetraneura nigriabdominalis*^a^	96.05	93	0.0
17A	*Homo sapiens*	99.54	99	0.0

^a^*Acarus farris*, *Tyrophagus putrescentiae*, *Dermestes coarctatus*, and *Tetraneura nigriabdominalis* are regarded as common stored product pests

### Mini-barcode performance in silico

Compared with the “standard” COI barcoding region, the mini-barcode LCO1490/HCO1777 was first validated in silico for Mantis. The phylogenetic reconstruction results showed that the topology of the NJ tree generated by the 232 bp region (Fig. S1A) was identical to that based on the 658 bp amplicon (Fig. S1B). Moreover, sequences of each species clustered into single lineages separately, and the species level discrimination ability of this short marker was acceptable for Mantis species.

### Sequence data analysis

In this study, 18 samples were amplified and sequenced successfully (Fig. S2). A total of 3,818,691 raw paired-end reads were generated from 18 samples, resulting in 3,110,601 quality-filtered reads after reads merging and primer trimming. Within 56 identified ASVs, 2 ASVs containing stop codons were removed. Finally, 37 Mantis ASVs (413,433 reads) and 17 ASVs from storage pests (743,579 reads) were retained as Supplementary Table S4. The reads count available for each sample and Mantis reads coverage were shown in Supplementary Table S5. Rarefaction curves were generated based on the number of reads for the mantis ASVs (Fig. S3).

### Species level taxon identification

To infer the taxonomic assignment of 37 Mantis ASVs, a similarity-based identification procedure was firstly performed. Not surprisingly, 13 and 7 ASVs were respectively determined as *Hierodula patellifera* and *Tenodera sinensis*, i.e., the certified Mantidis Ootheca described in China Pharmacopoeia. Besides, one ASV was determined as *Tenodera angustipennis*, and the other 16 ASVs could not be assigned to species due to the similarity values less than 98%.

**Fig. 2:**
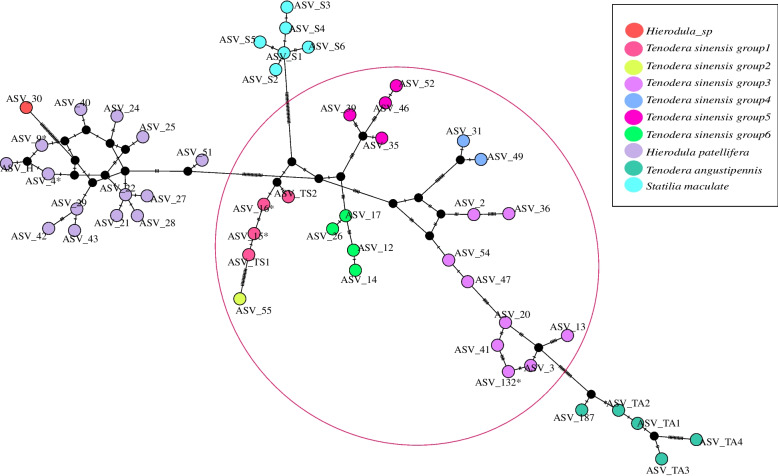
Haplotype networks for the mantis ASVs and bPTP MOTUs. Each MOTU was represented by a color. The haplotypes in the circles are considered to belong to *Tenodera sinensis*. (Lines linking haplotypes indicate the evolutionary paths among haplotypes, and vertical bars on the linking lines represent the mutation steps between haplotypes.). * ASV_4 and ASV_9 were 100% matched to *Hierodula patellifera* (KX611803.1 MW085419.1 NC_034283.1 MT439617.1). ASV_15 and ASV_16 were 100% matched to *Tenodera sinensis* (MN447996.1 MK829299.1), ASV_132 was 100% matched to *Tenodera angustipennis* (MZ049121.1)

For all haplotypes with exact or ambiguous taxon name revealed from Mantidis Ootheca samples, species delimitation methods were further applied to discover species level biodiversity. The ABGD method clustered 37 Mantis ASVs into 8 molecular operational taxonomic units (MOTUs). Meanwhile, the bPTP result was the same as ABGD, except that 8 ASVs were clustered into a single MOTU (MOTU_7) by ABGD while they were clustered by bPTP into MOTU_7 and MOTU_8. Together with the public data of *Tenodera sinensis*, *Statilia maculate*, *Hierodula patellifera*, and *Tenodera angustipennis*, the haplotype network map (Fig. 2**)** showed the genetic relationship of the COI haplotypes revealed in this study, and the putative bPTP MOTUs were highlighted. The correspondence between markers and public data in the haplotype network map is shown in Table S3.

Combining the results of BLAST-MEGAN identification, species definition and haplotype network analysis, besides bPTP MOTU_1 (*Hierodula patellifera*) and MOTU_9 (*Tenodera angustipennis*), cryptic MOTU_2 identified as *Hierodula_sp* was found around the *Hierodula patellifera* on the haplotype network map. At the intraspecies level, MOTU_3 - MOTU_8 consisting of 22 ASVs were identified as *Tenodera sinensis group* 1 - *Tenodera sinensis group* 6, separately. In addition, there are 14 different haplotypes belonging to *Hierodula patellifera*, suggesting the existence of complex intraspecific biodiversity. Finally, our taxonomic identification results containing 37 ASVs were shown in Table S6.

### Comparison of identification results between 18 samples

The information of 4580 Mantidis Ootheca individuals from the 18 samples and the identification results were in Table 1. In the results of Mantidis Ootheca original species identification, *Hierodula patellifera*, *Hierodula_sp* and *Tenodera sinensis* were identified in all 18 samples. *Tenodera angustipennis* was only identified in 3 samples (spx01, spx13 and spx14). The storage pests in each sample were mainly the insects of Coleoptera, Diptera, Hymenoptera and Mesostigmata. Specifically, *Stegobium_sp, Trogoderma variabile*, Carabidae_sp Chrysomelidae_sp1, Chrysomelidae_sp2 and *Lasioderma serricorne* were identified in all samples as storage pests (Table S7). Combining the results of DNA barcoding and DNA metabarcoding, our identification revealed only some of the pest species in the samples.

**Fig. 3:**
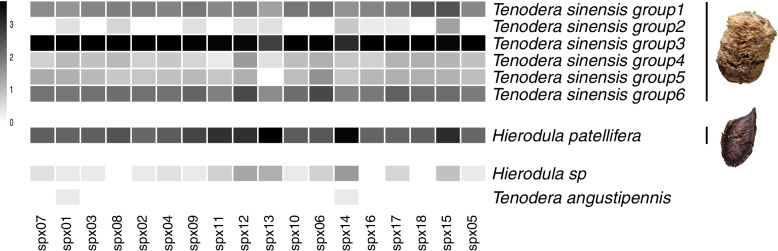
Heat map of mantis species identified in each of the 18 samples. Calculate the log10 value of the sequencing data reads for normalization and plot the heat map. The sequencing data reads were transformed log10 values and visualized by the heat map using R (pheatmap package). The numbers and color scale in the legend indicate the size of the transformed values. Darker indicates a higher abundance for that mantis species. The pictures of Mantidis Ootheca corresponding to the mantis species (*Tenodera sinensis*: Tuanpiaoxiao; *Hierodula patellifera*: Heipiaoxiao)

The abundance of mantis species identified in each of the 18 samples is shown in Fig. 3, Table S8 and Table S9. Two samples (spx13 and spx14) of Mantidis Ootheca original species were mainly identified as *Hierodula patellifera* (Heipiaoxiao). And the remaining 16 samples of Mantidis Ootheca original species were mainly identified as *Tenodera sinensis_group3* (Tuanpiaoxiao).

## Discussion

It is assumed that there is a complex biological composition in wild Chinese medicines [26, 27]. Previous research has indicated misidentifications due to the great variety of Mantis Ootheca available which are morphologically similar [5]. Especially, since the taxonomy of Mantis larvae has not yet been thoroughly studied, the conventional identification method depending on macroscopical characters has limitations on Mantis Ootheca. Moreover, the integrity of samples would be occasionally damaged during harvesting, processing and transportation, as the appearance of our samples 12A and 17A, which make the identification results questionable.

DNA barcoding provides an operational framework for species identification and cryptic biodiversity discovery. However, the identification results of natural animal-based medicine materials would be susceptible to interference from storage pests and DNA degradation. In this study, five amplicon sequences of the ten Mantidis Ootheca representative samples were identified as common stored product pests. On the other hand, it is reported that mixed oothecae from different species within one package are currently sold in commercial markets [28]. In this study, DNA metabarcoding was used for the identification of origin species in a large amount of wild Chinese medicines, which were affected by storage pests. Within 4580 individuals we investigated, one cryptic species was recovered, besides 3 species as genuine or adulterants of Mantidis Ootheca reported before. Meanwhile, 37 Mantis ASVs were also obtained, while 14 of them were regarded as belonging to *Hierodula patellifera*, and 22 ASVs belonging to six genetic groups within *Tenodera sinensis*. It remains to be clarified whether these above-mentioned interspecific and intraspecific diversity influence the efficacy of this kind of natural medicine.

The high-throughput sequencing technology used for DNA metabarcoding produces a large number of parallel sequencing reads, making it possible to analyze the biological composition of complex samples. Adversely, even the slightest existence of exogenous DNA contamination may be detected and can potentially further complicate the interpretation of the results. In this study, the laboratory environment and every step of the experimental operation were strictly controlled, and the gels showed no bands for the negative control. Nevertheless, considering the super-sensitivity of high-throughput sequencing, the negative control should be sequenced as well to further provide the background of potential contamination among samples, and this protocol should be adopted by other similar works in future.

## Conclusions

In this study, 18 DNA samples, a total of 4580 commercially available Mantidis Ootheca individuals with disturbance of storage pests, were identified using DNA metabarcoding. 37 Mantis ASVs and 9 Mantis MOTUs were identified through species delimitation, and the intraspecific diversity was depicted as haplotype network plot. Besides *Tenodera sinensis* and *Hierodula patellifera* as genuine sources defined in the Chinese Pharmacopoeia, *Tenodera angustipennis* was also the origin species of Mantidis Ootheca. In summary, as exemplified by the Mantidis Ootheca, DNA metabarcoding technology will make more contributions to improving the identification system of TCM and improve the quality level of TCM.

## Supplementary Information

**Additional file 1: Table S1.** The morphological characteristics information of ten Mantidis Ootheca representative. **Table S2.** Primer tag sequence. **Table S3.** Mantidis COI records from GenBank. **Table S4.** 54 ASVs and Blast-Megan results. **Table S5.** Number of reads available for each sample. **Table S6.** Taxonomic identification information of 37 ASVs. **Table S7.** Sequence readings of pest species*. **Table S8.** Sequence readings of mantis species before rarefied*. **Table S9.** Sequence readings of mantis species after rarefied*. **Fig. S1.** The phylogeny of Mantis COI sequences downloaded from NCBI. A. The neighbor-joining tree based on 232 bp fragment trimmed by LCO1490/HCO1777. B. The neighbor-joining tree based on 658 bp fragment trimmed by LCO1490/HCO2198. **Fig. S2.** The gel electrophoresis diagram of the PCR products of 18 samples. From left to right: DL500 marker, 01–18 were samples spx01-spx18 respectively, K and F were negative controls during the experiment. **Fig. S3.** Rarefaction curves of mantis ASVs in each of the 18 samples. Different colors indicate different samples. Solid lines indicate actual sampling, dashed lines indicate predicted sampling.

## Data Availability

The Illumina datasets generated for this study can be found in the NCBI Sequence Read Archive (SRA) accession numbers: PRJNA843927.

## References

[1] Yang S. The divine farmer's materia medica: a translation of the Shen Nong Ben Cao Jing[M]. Blue Poppy Enterprises, Inc.; 1998.

[2] Ryu SM, Nam H-H, Kim JS, Song J-H, Seo YH, Kim HS, Lee AY, Kim WJ, Lee D, Moon BC (2021). Chemical constituents of the egg cases of Tenodera Angustipennis (Mantidis Ootheca) with intracellular reactive oxygen species scavenging activity. Biomolecules.

[3] Committee, N.P (2015). Pharmacopoeia of the People’s Republic of China. Part.

[4] Ge D, Chen X. Advances of research on the Mantodea from China. J Mountain Agric Biol. 2004;23(6):525–8.

[5] Wen L-L, Wan D-G, Ren Y, Li J-D, Guo J-L (2013). Corresponding relationship between Mantis and Mantidis Oötheca (Sangpiaoxiao). Zhongguo Zhong Yao Za Zhi.

[6] Wang X, Hou F, Wang Y, Wang Y, Li J, Yuan Y, Peng C, Guo J (2015). Identification of original species of Mantidis Oötheca (Sangpiaoxiao) based on DNA barcoding. Zhongguo Zhong Yao Za Zhi.

[7] Liu Q-P, Liu Z-J, Wang G-L, Yin Z-X (2021). Taxonomic revision of the praying Mantis subfamily Hierodulinae of China (Mantodea: Mantidae). Zootaxa.

[8] Tan Z, Lei Y, Zhang B, Huang L (1997). Comparison of pharmacological studies on ootheca Mantidis. Zhongguo Zhong Yao Za Zhi.

[9] Saccò M, Guzik MT, van der Heyde M, Nevill P, Cooper SJB, Austin AD, Coates PJ, Allentoft ME, White NE (2022). EDNA in subterranean ecosystems: applications, technical aspects, and future prospects. Sci Total Environ.

[10] Kulik T, Bilska K, Żelechowski M (2020). Promising perspectives for detection, identification, and quantification of plant pathogenic Fungi and oomycetes through targeting mitochondrial DNA. Int J Mol Sci.

[11] Yang F, Ding F, Chen H, He M, Zhu S, Ma X, Jiang L, Li H (2018). DNA barcoding for the identification and authentication of animal species in traditional medicine. Evid Based Complement Alternat Med.

[12] Vesterinen EJ, Ruokolainen L, Wahlberg N, Peña C, Roslin T, Laine VN, Vasko V, Sääksjärvi IE, Norrdahl K, Lilley TM (2016). What you need is what you eat? Prey selection by the bat Myotis Daubentonii. Mol Ecol.

[13] Folmer O, Black M, Hoeh W, Lutz R, Vrijenhoek R (1994). DNA primers for amplification of mitochondrial cytochrome c oxidase subunit I from diverse metazoan invertebrates. Mol Mar Biol Biotechnol.

[14] Andújar C, Arribas P, Yu DW, Vogler AP, Emerson BC (2018). Why the COI barcode should be the community DNA Metabarcode for the Metazoa. Mol Ecol.

[15] Elbrecht V, Braukmann TWA, Ivanova NV, Prosser SWJ, Hajibabaei M, Wright M, Zakharov EV, Hebert PDN, Steinke D (2019). Validation of COI Metabarcoding primers for terrestrial arthropods. PeerJ.

[16] Brown DS, Jarman SN, Symondson WOC (2012). Pyrosequencing of prey DNA in reptile Faeces: analysis of earthworm consumption by slow Worms. Mol Ecol Resour.

[17] Bolger AM, Lohse M, Usadel B (2014). Trimmomatic: a flexible trimmer for Illumina sequence data. Bioinformatics.

[18] Aronesty E (2013). Comparison of sequencing utility programs. Open Bioinformatics J.

[19] Bushnell B, Rood J, Singer E (2017). BBMerge - accurate paired shotgun read merging via overlap. Plos One.

[20] Caporaso JG, Kuczynski J, Stombaugh J, Bittinger K, Bushman FD, Costello EK, Fierer N, Peña AG, Goodrich JK, Gordon JI (2010). QIIME allows analysis of high-throughput community sequencing data. Nat Methods.

[21] Edgar RC (2013). UPARSE: highly accurate OTU sequences from microbial amplicon reads. Nat Methods.

[22] Edgar RC (2010). Search and clustering orders of magnitude faster than BLAST. Bioinformatics.

[23] Altschul SF, Madden TL, Schäffer AA, Zhang J, Zhang Z, Miller W, Lipman DJ (1997). Gapped BLAST and PSI-BLAST: a new generation of protein database search programs. Nucleic Acids Res.

[24] Huson DH, Auch AF, Qi J, Schuster SC (2007). MEGAN analysis of metagenomic data. Genome Res.

[25] Zhang X, Wang W, Yu X, Liu Y, Li W, Yang H, Cui Y, Tian X (2022). Biological composition analysis of a natural medicine, Faeces Vespertilionis, with complex sources using DNA Metabarcoding. Sci Rep.

[26] Wang C, Zhang Y, Ding H, Song M, Yin J, Yu H, Li Z, Han L, Zhang Z (2021). Authentication of Zingiber species based on analysis of metabolite profiles. Front Plant Sci.

[27] Zhu S, Li Q, Chen S, Wang Y, Zhou L, Zeng C, Dong J (2018). Phylogenetic analysis of Uncaria species based on internal transcribed spacer (ITS) region and ITS2 secondary structure. Pharm Biol.

[28] Song J-H, Cha J-M, Moon BC, Kim WJ, Yang S, Choi G (2020). Mantidis Oötheca (Mantis egg case) original species identification via morphological analysis and DNA barcoding. J Ethnopharmacol.

