# Supplementary material for: Species diversity analysis of commercial Mantidis Ootheca samples contaminated by store pests based on DNA metabarcoding
Source: BMC Genomics. 2022 Oct 21;23:720. doi: 10.1186/s12864-022-08955-1 (PMC9587553; doi:10.1186/s12864-022-08955-1)
Supplement: Supplementary file 1 — Additional file 1: Table S1. The morphological characteristics information of ten Mantidis Ootheca representative. Table S2. Primer tag sequence. Table S3. Mantidis COI records from GenBank. Table S4. 54 ASVs and Blast-Megan results. Table S5. Number of reads available for each sample. Table S6. Taxonomic identification information of 37 ASVs. Table S7. Sequence readings of pest species*. Table S8. Sequence readings of mantis species before rarefied*. Table S9. Sequence readings of mantis species after rarefied*. Fig. S1. The phylogeny of Mantis COI sequences downloaded from NCBI. A. The neighbor-joining tree based on 232 bp fragment trimmed by LCO1490/HCO1777. B. The neighbor-joining tree based on 658 bp fragment trimmed by LCO1490/HCO2198. Fig. S2. The gel electrophoresis diagram of the PCR products of 18 samples. From left to right: DL500 marker, 01–18 were samples spx01-spx18 respectively, K and F were negative controls during the experiment. Fig. S3. Rarefaction curves of mantis ASVs in each of the 18 samples. Different colors indicate different samples. Solid lines indicate actual sampling, dashed lines indicate predicted sampling. [file 12864_2022_8955_MOESM1_ESM.docx]

## Supplementary Material

## Supplementary Tables

Table S1. The morphological characteristics information of ten Mantidis Ootheca representative samples

| Sample ID | Shape | Color |
| --- | --- | --- |
| SPX6-A | fusiform | brown |
| SPX6-B | barrel-like | brown |
| SPX12-A | fusiform | brown |
| SPX12-B | barrel-like | brown |
| SPX13-A | ellipsoid | reddish |
| SPX13-B | barrel-like | brown |
| SPX14-A | fusiform | brown |
| SPX15-A | barrel-like | brown |
| SPX15-B | ellipsoid | black |
| SPX17-A | fusiform | black |

Table S2. Primer tag sequence

| tag | Tag sequence |
| --- | --- |
| 1 | TCGTAGCA |
| 2 | TGAGACGT |
| 3 | TGCTCACT |
| 4 | CAGCTGAG |
| 5 | ATACGCTC |
| 6 | CTGTCTCG |
| 7 | ATAGAGCG |
| 8 | ATCACTGC |
| 9 | ACTAGCAG |
| 10 | CATGCTGT |
| 11 | CTCATGCT |
| 12 | CTCTACGA |
| 13 | CTAGTAGC |
| 14 | AGCGTATG |
| 15 | AGATCGAC |
| 16 | AGTCTGCA |
| 17 | ACGCGAGA |
| 18 | ACGATGTC |

Table S3. Mantidis COI records from GenBank

| Species | Accession number | Each haplotype label in the haplotype network map |
| --- | --- | --- |
| *Tenodera sinensis* | KT036561.1 | ASV_TS1 |
|  | MT129400.1  MN447996.1 | ASV_TS2  ASV_15 |
|  | MK829299.1 | ASV_16 |
| *Tenodera angustipennis* | MT129147.1 | ASV_TA1 |
|  | MT129420.1 | ASV_TA2 |
|  | MW085479.1 | ASV_TA3 |
|  | MT129197.1  MZ049121.1 | ASV_TA4  ASV_132 |
| *Hierodula patellifera* | MT129374.1 | ASV_H |
|  | KX611803.1 | ASV_4 |
|  | MW085419.1 | ASV_4 |
|  | NC_034283.1  MT439617.1 | ASV_4  ASV_9 |
| *Statilia maculata* | KM362717.1 | ASV_S1 |
|  | KM362718.1 | ASV_S1 |
|  | KM362722.1 | ASV_S1 |
|  | KM362720.1 | ASV_S1 |
|  | MW085640.1 | ASV_S1 |
|  | KM362723.1 | ASV_S2 |
|  | KT036560.1 | ASV_S3 |
|  | NC_056840.1 | ASV_S4 |
|  | KM362721.1 | ASV_S5 |
|  | KM362719.1 | ASV_S6 |

Table S4. 54 ASVs and Blast-Megan results

| ASV_ID | Best Blast Results | Percent Identity (%) |
| --- | --- | --- |
| ASV_4 | *Hierodula patellifera* | 100 |
| ASV_9 | *Hierodula patellifera* | 100 |
| ASV_21 | *Hierodula patellifera* | 99 |
| ASV_22 | *Hierodula patellifera* | 99 |
| ASV_27 | *Hierodula patellifera* | 99 |
| ASV_28 | *Hierodula patellifera* | 99 |
| ASV_24 | *Hierodula patellifera* | 98 |
| ASV_25 | *Hierodula patellifera* | 98 |
| ASV_29 | *Hierodula patellifera* | 98 |
| ASV_40 | *Hierodula patellifera* | 98 |
| ASV_43 | *Hierodula patellifera* | 98 |
| ASV_51 | *Hierodula patellifera* | 98 |
| ASV_42 | *Hierodula patellifera* | 97 |
| ASV_2 | *Tenodera sinensis* | 94 |
| ASV_3 | *Tenodera sinensis* | 99 |
| ASV_132 | *Tenodera sinensis* | 100 |
| ASV_20 | *Tenodera sinensis* | 97 |
| ASV_36 | *Tenodera sinensis* | 93 |
| ASV_41 | *Tenodera sinensis* | 98 |
| ASV_15 | *Tenodera sinensis* | 100 |
| ASV_16 | *Tenodera sinensis* | 100 |
| ASV_17 | *Tenodera sinensis* | 98 |
| ASV_26 | *Tenodera sinensis* | 98 |
| ASV_35 | *Mantidae sp. DIMC031-09* | 97 |
| ASV_12 | *Tenodera sinensis* | 97 |
| ASV_39 | *Mantidae sp. DIMC031-09* | 96 |
| ASV_46 | *Mantidae sp. DIMC031-09* | 96 |
| ASV_52 | *Mantidae sp. DIMC031-09* | 96 |
| ASV_14 | *Tenodera sinensis* | 97 |
| ASV_55 | *Tenodera sinensis* | 95 |
| ASV_47 | *Tenodera sinensis* | 96 |
| ASV_13 | *Tenodera sinensis* | 97 |
| ASV_30 | *Hierodula patellifera* | 92 |
| ASV_54 | *Tenodera sinensis* | 95 |
| ASV_31 | *Tenodera sinensis* | 92 |
| ASV_49  ASV_187 | *Tenodera sinensis*  *Tenodera angustipennis* | 92  99 |
| ASV_10 | *Argentinatachoides balli* | 91 |
| ASV_11 | *Trogoderma variabile* | 99 |
| ASV_136 | *Trogoderma variabile* | 100 |
| ASV_167 | *Stegobium paniceum* | 100 |
| ASV_18 | *Lasioderma serricorne* | 100 |
| ASV_19 | *Stegobium paniceum* | 94 |
| ASV_33 | *Dicladispa armigera* | 88 |
| ASV_34 | *Blattisocius tarsalis* | 99 |
| ASV_37 | *Blattisocius tarsalis* | 98 |
| ASV_38 | *Tribolium castaneum* | 100 |
| ASV_44 | *Tribolium castaneum* | 100 |
| ASV_45 | *Anthrenus scrophulariae* | 88 |
| ASV_48 | *Podagrion bouceki* | 91 |
| ASV_5 | *Trogoderma variabile* | 100 |
| ASV_50 | *Tydeidae sp* | 87 |
| ASV_53 | *Trogoderma variabile* | 97 |
| ASV_88 | Chrysomelidae sp. | 86 |

Table S5. Number of reads available for each sample

| Sample ID | Total reads | Reads determined as Mantis | Coverage (Mantis reads per individual) |
| --- | --- | --- | --- |
| spx01 | 17,095 | 12,198 | 76.24 |
| spx02 | 23,815 | 19,731 | 82.21 |
| spx03 | 82,827 | 15,771 | 39.33 |
| spx04 | 110,805 | 31,351 | 125.40 |
| spx05 | 65,813 | 39,713 | 93.88 |
| spx06 | 25,215 | 23,173 | 66.59 |
| spx07 | 91,632 | 40,207 | 155.84 |
| spx08 | 50,016 | 11,536 | 33.34 |
| spx09 | 68,638 | 8,698 | 29.19 |
| spx10 | 32,296 | 28,109 | 119.61 |
| spx11 | 81,248 | 5,239 | 15.14 |
| spx12 | 27,935 | 11,761 | 44.89 |
| spx13 | 111,815 | 40,290 | 110.38 |
| spx14 | 119,999 | 32,809 | 218.73 |
| spx15 | 135,279 | 17,551 | 117.01 |
| spx16 | 77,340 | 44,539 | 291.10 |
| spx17 | 9,792 | 8,762 | 74.89 |
| spx18 | 25,452 | 21,995 | 281.99 |

Table S6 taxonomic identification information of 37 ASVs

| ASV_ID | Best GenBank Blast Results | Percent Identity (%) | ABGD MOTU_ID | bPTP MOTU_ID | Final identification result |
| --- | --- | --- | --- | --- | --- |
| ASV_21 | *Hierodula patellifera* | 99 | 1 | 1 | *Hierodula patellifera* |
| ASV_22 | *Hierodula patellifera* | 99 |  |  |  |
| ASV_24 | *Hierodula patellifera* | 98 |  |  |  |
| ASV_25 | *Hierodula patellifera* | 98 |  |  |  |
| ASV_27 | *Hierodula patellifera* | 99 |  |  |  |
| ASV_28 | *Hierodula patellifera* | 99 |  |  |  |
| ASV_29 | *Hierodula patellifera* | 98 |  |  |  |
| ASV_4 | *Hierodula patellifera* | 100 |  |  |  |
| ASV_40 | *Hierodula patellifera* | 98 |  |  |  |
| ASV_43 | *Hierodula patellifera* | 98 |  |  |  |
| ASV_51 | *Hierodula patellifera* | 98 |  |  |  |
| ASV_9 | *Hierodula patellifera* | 100 |  |  |  |
| ASV_42 | *Hierodula patellifera* | 98 |  |  |  |
| ASV_30 | *Hierodula patellifera* | 92 | 2 | 2 | *Hierodula_sp* |
| ASV_15 | *Tenodera sinensis* | 100 | 3 | 3 | *Tenodera sinensis group1* |
| ASV_16 | *Tenodera sinensis* | 100 |  |  |  |
| ASV_55 | *Tenodera sinensis* | 95 | 4 | 4 | *Tenodera sinensis group2* |
| ASV_13 | *Tenodera sinensis* | 97 | 5 | 5 | *Tenodera sinensis group3* |
| ASV_132 | *Tenodera sinensis* | 100 |  |  |  |
| ASV_2 | *Tenodera sinensis* | 94 |  |  |  |
| ASV_20 | *Tenodera sinensis* | 97 |  |  |  |
| ASV_3 | *Tenodera sinensis* | 99 |  |  |  |
| ASV_36 | *Tenodera sinensis* | 93 |  |  |  |
| ASV_41 | *Tenodera sinensis* | 98 |  |  |  |
| ASV_47 | *Tenodera sinensis* | 96 |  |  |  |
| ASV_54 | *Tenodera sinensis* | 95 |  |  |  |
| ASV_31 | *Tenodera sinensis* | 92 | 6 | 6 | *Tenodera sinensis group4* |
| ASV_49 | *Tenodera sinensis* | 92 |  |  |  |
| ASV_35 | *Mantidae sp. DIMC031-09* | 97 | 7 | 7 | *Tenodera sinensis group5* |
| ASV_39 | *Mantidae sp. DIMC031-09* | 96 |  |  |  |
| ASV_46 | *Mantidae sp. DIMC031-09* | 96 |  |  |  |
| ASV_52 | *Mantidae sp. DIMC031-09* | 96 |  |  |  |
| ASV_12 | *Tenodera sinensis* | 97 |  | 8 | *Tenodera sinensis group6* |
| ASV_14 | *Tenodera sinensis* | 97 |  |  |  |
| ASV_17 | *Tenodera sinensis* | 98 |  |  |  |
| ASV_26 | *Tenodera sinensis* | 98 |  |  |  |
| ASV_187 | *Tenodera angustipennis* | 99 | 8 | 9 | *Tenodera angustipennis* |

Table S7 Sequence readings of pest species*

| Species | spx07 | spx01 | spx03 | spx08 | spx02 | spx04 | spx09 | spx11 | spx12 | spx13 | spx10 | spx06 | spx14 | spx16 | spx17 | spx18 | spx15 | spx05 |
| --- | --- | --- | --- | --- | --- | --- | --- | --- | --- | --- | --- | --- | --- | --- | --- | --- | --- | --- |
| *Blattisocius_tarsalis* | 99 | 2 | 2 | 45 | 5 | 32 | 302 | 1 | 13 | 6 | 1 | 7 | 19 | 4 | 1 | 0 | 3 | 11 |
| *Stegobium_sp* | 390 | 59 | 185 | 303 | 91 | 438 | 21631 | 204 | 151 | 277 | 197 | 167 | 312 | 263 | 49 | 106 | 338 | 185 |
| *Tribolium_castaneum* | 3 | 0 | 5 | 11 | 1 | 11 | 128 | 31 | 116 | 13 | 10 | 50 | 18 | 4 | 0 | 1 | 2 | 9 |
| *Trogoderma_variabile* | 1097 | 249 | 719 | 420 | 470 | 1017 | 399 | 73974 | 3832 | 925 | 1029 | 540 | 744 | 1140 | 191 | 332 | 957 | 945 |
| *Dermestidae_sp1* | 15 | 1 | 9 | 8 | 0 | 28 | 2 | 0 | 1 | 13 | 1 | 0 | 15 | 16 | 0 | 0 | 24 | 13 |
| *Carabidae_sp* | 148 | 19 | 152 | 105 | 34 | 233 | 4898 | 32 | 46 | 156 | 61 | 65 | 242 | 132 | 8 | 42 | 227 | 64 |
| *Chrysomelidae_sp1* | 2645 | 268 | 3392 | 1953 | 232 | 4064 | 1758 | 188 | 4434 | 3429 | 316 | 107 | 4255 | 1793 | 109 | 221 | 32903 | 1282 |
| *Chrysomelidae_sp2* | 46994 | 4291 | 62575 | 34804 | 3237 | 73589 | 30575 | 1569 | 7565 | 66594 | 2553 | 1069 | 81532 | 29417 | 557 | 2747 | 83236 | 23559 |
| *Lasioderma_serricorne* | 20 | 5 | 2 | 796 | 8 | 15 | 237 | 10 | 14 | 111 | 19 | 15 | 52 | 25 | 2 | 6 | 35 | 28 |
| *Torymidae_sp* | 10 | 3 | 14 | 33 | 4 | 25 | 8 | 0 | 2 | 1 | 0 | 22 | 0 | 5 | 4 | 0 | 3 | 3 |
| *Tydeidae_sp* | 4 | 0 | 1 | 2 | 2 | 2 | 2 | 0 | 0 | 0 | 0 | 0 | 1 | 2 | 109 | 2 | 0 | 1 |

* The number indicates the number of reads of this species in the sample.

Table S8 Sequence readings of mantis species before rarefied*

| Species | spx07 | spx01 | spx03 | spx08 | spx02 | spx04 | spx09 | spx11 | spx12 | spx13 | spx10 | spx06 | spx14 | spx16 | spx17 | spx18 | spx15 | spx05 |
| --- | --- | --- | --- | --- | --- | --- | --- | --- | --- | --- | --- | --- | --- | --- | --- | --- | --- | --- |
| *Hierodula_patellifera* | 1624 | 404 | 603 | 690 | 597 | 1481 | 773 | 956 | 2058 | 35278 | 1110 | 1162 | 24927 | 1464 | 305 | 829 | 3588 | 1287 |
| *Hierodula_sp* | 14 | 2 | 3 | 2 | 8 | 15 | 3 | 4 | 41 | 133 | 5 | 12 | 183 | 13 | 3 | 1 | 30 | 8 |
| *Tenodera_sinensis_group1* | 428 | 76 | 183 | 184 | 223 | 348 | 144 | 52 | 156 | 211 | 472 | 452 | 210 | 511 | 117 | 971 | 1134 | 398 |
| *Tenodera_sinensis_group2* | 4 | 2 | 1 | 5 | 0 | 2 | 4 | 0 | 3 | 8 | 1 | 2 | 9 | 7 | 1 | 2 | 66 | 3 |
| *Tenodera_sinensis_group3* | 37051 | 11461 | 14597 | 10358 | 18447 | 28612 | 7444 | 4152 | 8498 | 4261 | 25913 | 19198 | 7096 | 41614 | 7999 | 19570 | 12222 | 37191 |
| *Tenodera_sinensis_group4* | 29 | 23 | 24 | 17 | 20 | 40 | 5 | 1 | 72 | 7 | 24 | 70 | 13 | 64 | 20 | 45 | 21 | 34 |
| *Tenodera_sinensis_group5* | 80 | 19 | 29 | 14 | 28 | 59 | 15 | 6 | 30 | 30 | 28 | 168 | 39 | 60 | 18 | 39 | 37 | 69 |
| *Tenodera_sinensis_group6* | 977 | 210 | 331 | 266 | 408 | 794 | 310 | 68 | 903 | 361 | 556 | 2109 | 331 | 806 | 299 | 538 | 453 | 723 |
| *Tenodera_angustipennis* | 0 | 1 | 0 | 0 | 0 | 0 | 0 | 0 | 0 | 1 | 0 | 0 | 1 | 0 | 0 | 0 | 0 | 0 |

* The number indicates the number of reads of this species in the sample.

Table S9 Sequence readings of mantis species after rarefied*

| Species | spx07 | spx01 | spx03 | spx08 | spx02 | spx04 | spx09 | spx11 | spx12 | spx13 | spx10 | spx06 | spx14 | spx16 | spx17 | spx18 | spx15 | spx05 |
| --- | --- | --- | --- | --- | --- | --- | --- | --- | --- | --- | --- | --- | --- | --- | --- | --- | --- | --- |
| *Tenodera_sinensis_group1* | 47 | 34 | 58 | 73 | 75 | 63 | 90 | 52 | 64 | 22 | 92 | 105 | 36 | 56 | 74 | 237 | 308 | 52 |
| *Tenodera_sinensis_group2* | 0 | 2 | 0 | 3 | 0 | 0 | 2 | 0 | 2 | 2 | 0 | 0 | 5 | 1 | 1 | 0 | 21 | 0 |
| *Tenodera_sinensis_group3* | 4865 | 4916 | 4847 | 4724 | 4880 | 4827 | 4478 | 4152 | 3757 | 561 | 4787 | 4352 | 1135 | 4905 | 4777 | 4637 | 3686 | 4927 |
| *Tenodera_sinensis_group4* | 4 | 9 | 7 | 9 | 4 | 5 | 3 | 1 | 28 | 2 | 8 | 14 | 6 | 4 | 11 | 14 | 11 | 7 |
| *Tenodera_sinensis_group5* | 17 | 13 | 9 | 5 | 6 | 7 | 9 | 6 | 13 | 0 | 8 | 41 | 6 | 10 | 13 | 14 | 12 | 8 |
| *Tenodera_sinensis_group6* | 106 | 93 | 107 | 118 | 110 | 108 | 176 | 68 | 413 | 48 | 116 | 452 | 59 | 86 | 184 | 135 | 127 | 81 |
| *Hierodula_patellifera* | 198 | 170 | 210 | 307 | 163 | 227 | 480 | 956 | 944 | 4591 | 227 | 271 | 3963 | 177 | 176 | 202 | 1067 | 163 |
| *Hierodula_sp* | 2 | 1 | 1 | 0 | 1 | 2 | 1 | 4 | 18 | 13 | 1 | 4 | 28 | 0 | 3 | 0 | 7 | 1 |
| *Tenodera_angustipennis* | 0 | 1 | 0 | 0 | 0 | 0 | 0 | 0 | 0 | 0 | 0 | 0 | 1 | 0 | 0 | 0 | 0 | 0 |

* The number indicates the number of reads of this species in the sample. The data were rarefied in order to make them comparable between samples.

## Supplementary Figures


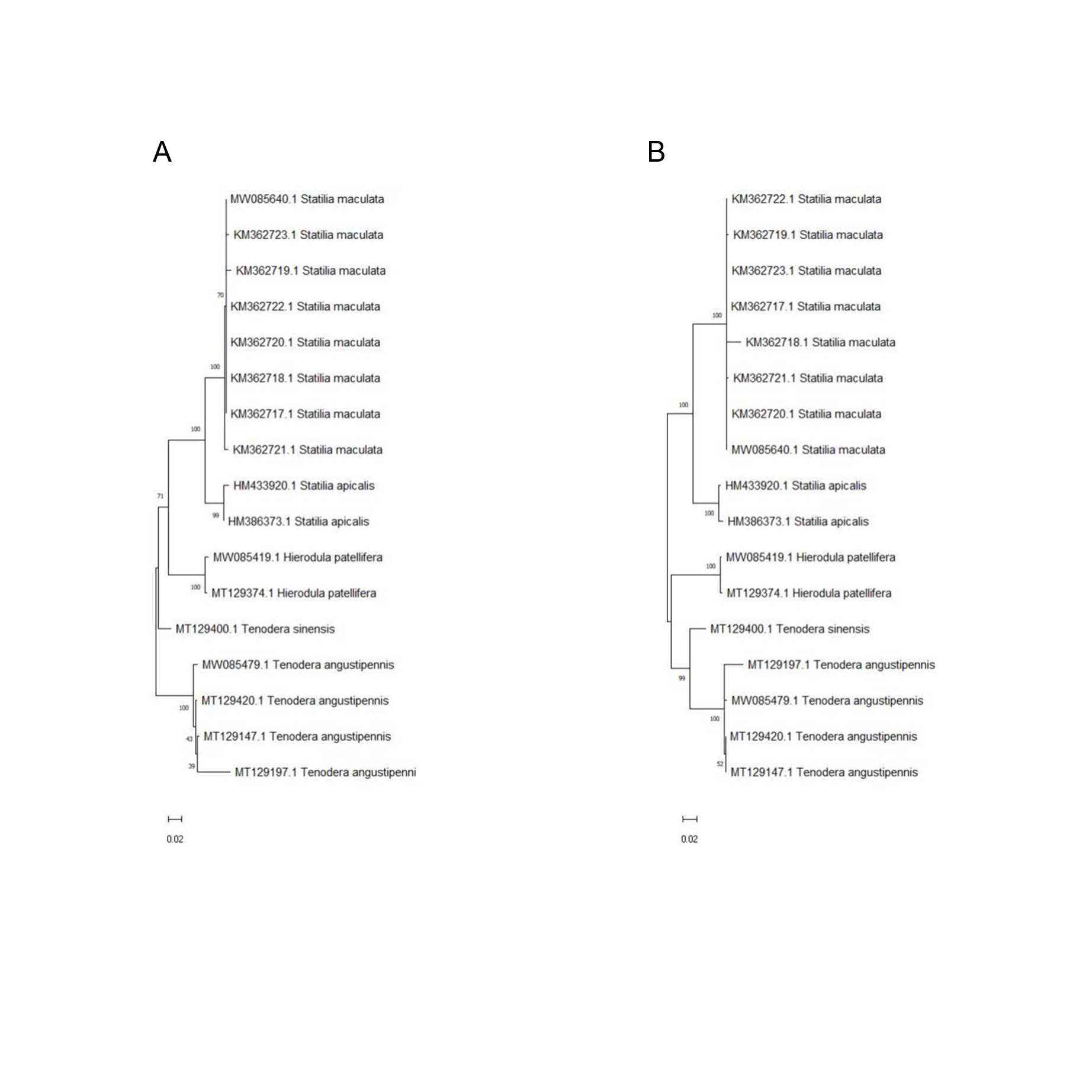


**Figure S1** The phylogeny of Mantis COI sequences downloaded from NCBI. A. The neighbor-joining tree based on 232 bp fragment trimmed by LCO1490/HCO1777. B. The neighbor-joining tree based on 658 bp fragment trimmed by LCO1490/HCO2198.


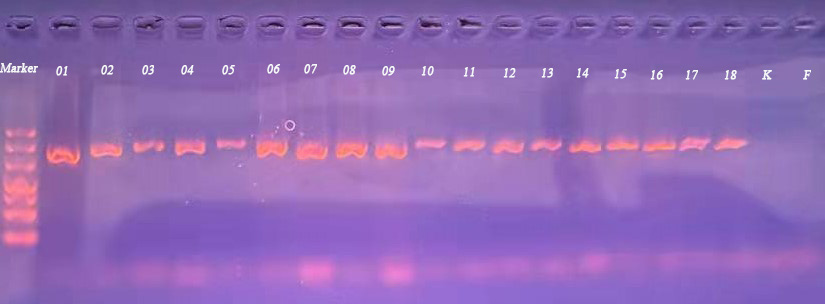


**Figure S2** The gel electrophoresis diagram of the PCR products of 18 samples. From left to right: DL500 marker, 01-18 were samples spx01-spx18 respectively, K and F were negative controls during the experiment.


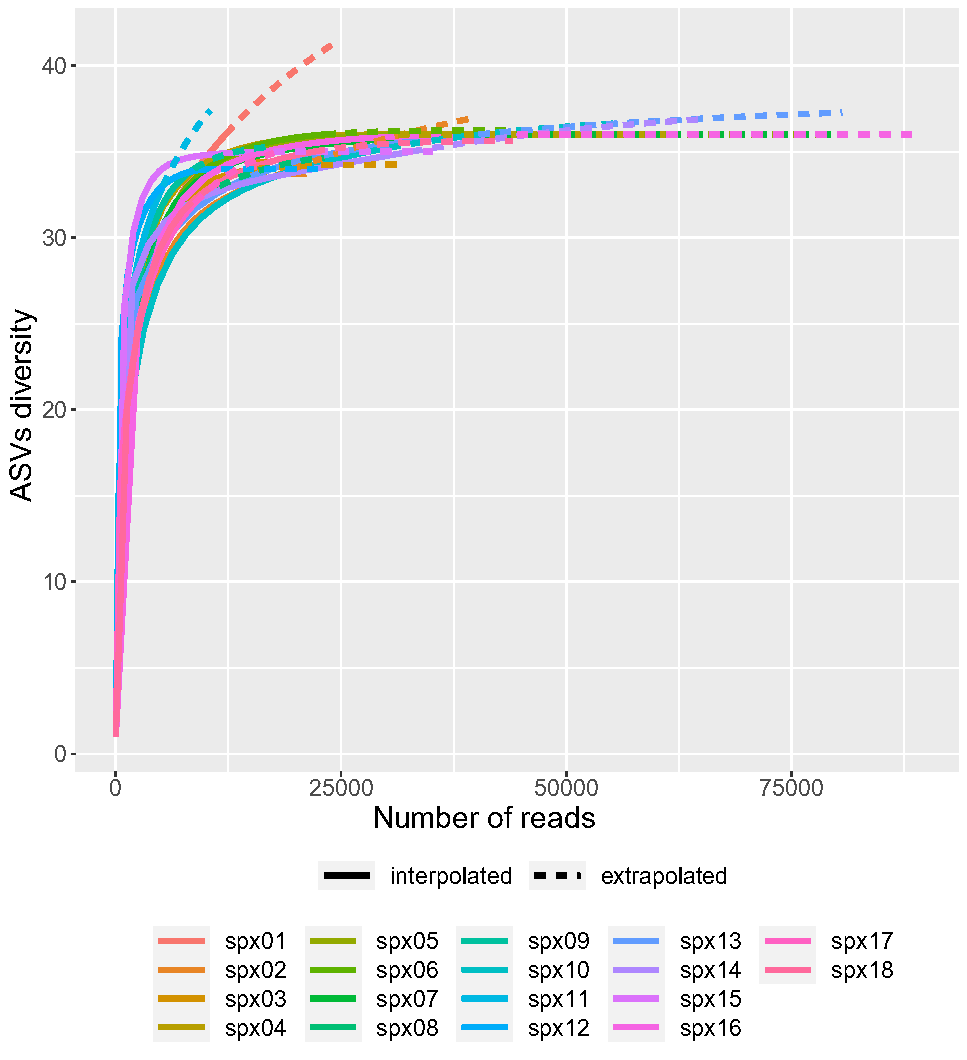


**Figure S3** Rarefaction curves of mantis ASVs in each of the 18 samples. Different colors indicate different samples. Solid lines indicate actual sampling, dashed lines indicate predicted sampling.
